# ^1^H NMR metabolomic profiling of resistant and susceptible oil palm root tissues in response to *Ganoderma boninense* at the nursery stage

**DOI:** 10.1038/s41598-025-01691-y

**Published:** 2025-05-14

**Authors:** Syarul Nugroho, Hernawan Yuli Rahmadi, Arfan Nazhri Simamora, Abdul Razak Purba

**Affiliations:** 1 Plant Breeding Research Group, Indonesian Oil Palm Research Institute, Jl. Brigjend Katamso No. 51, Medan, 20158 North Sumatera Indonesia; 2 PT Riset Perkebunan Nusantara, Jl. Salak No. 1A, Bogor, 16128 West Java Indonesia

**Keywords:** ^1^H NMR, Metabolomic profiling, Metabolic biomarkers, Oil palm resistance, Metabolomics, Metabolomics, Plant biotechnology, Plant breeding, Plant immunity, Plant stress responses

## Abstract

Oil palm plantations face serious challenges from *Ganoderma boninense*, a pathogen that causes basal stem rot (BSR), leading to significant productivity losses, with an estimated economic impact of 68.73%. *Ganoderma* spreads through direct root contact and airborne spores, affecting plantations across Indonesia, Malaysia, and other countries. Understanding the mechanisms of oil palm resistance to *Ganoderma* is crucial for developing effective strategies. Metabolomic profiling, ¹H NMR spectroscopy, offers a promising tool for identifying and quantifying metabolic changes associated with *Ganoderma* resistance. This study, ¹H NMR was employed to analyze root tissues of resistant, susceptible, and control oil palm seedlings exposed to *Ganoderma*. The results indicated that PCA effectively differentiated resistant palms from susceptible ones, while PLS-DA identified 14 significant metabolites. Further analysis using OPLS-DA and ROC revealed that ascorbic acid, D-gluconic acid, D-fructose, and 2-oxoisovalerate could serve as potential biomarkers for screening resistant palms. The metabolites identified in this study hold considerable promise for supporting breeding programs to develop oil palm varieties with enhanced resistance to BSR.

**Supplementary Information:**

The online version contains supplementary material available at 10.1038/s41598-025-01691-y.

## Introduction

Oil palm (*Elaeis guineensis*) is a vital crop cultivated in tropical regions within ± 10° latitude of the equator, including parts of Africa, Southeast Asia, and South and Central America. These regions offer favorable climatic conditions, such as high rainfall, for oil palm growth^1^. In Indonesia, oil palm has been commercially cultivated since 1911 under Dutch rule^2^. To date, Indonesia has been the largest exporter of palm oil in the global market for the past two decades^3^. The palm oil industry has become an important part of Indonesia’s economy, contributing to employment, rural development, and foreign exchange earnings.

Oil palm plantations face serious challenges from *Ganoderma boninense*, a pathogen responsible for causing basal stem rot (BSR). *Ganoderma* infects the base or middle of the oil palm trunk, leading to tissue decay and ultimately plant death^4^. The disease spreads rapidly through direct contact between infected and healthy roots, as well as via airborne basidiospores, which facilitate long-distance dispersal^5^. *Ganoderma* infections are not limited to Indonesia and Malaysia but have also been reported in several African countries and Colombia^6^. According to research by Kamu et al.^7^, economic losses due to Ganoderma infections are estimated to reach 68.73%.

No effective method for controlling BSR has been found, as technical cultural approaches, chemical control, and biological control have all been employed without success^8^. Breeding for oil palm resistance against *Ganoderma* offers a potential solution, but it remains challenging because disease resistance is controlled by multiple genes^9^. Developing multigenic resistance is complex, as resistance (R) genes are typically unlinked, making it challenging and labor-intensive to create and maintain R gene combinations in breeding programs^10^.

Understanding the mechanisms behind oil palm resistance to *Ganoderma* is essential for effectively managing this disease. Therefore, research exploring these resistance mechanisms, particularly at the metabolomic level, is highly promising, as it is closely linked to the phenotypic level^11^. Metabolomic profiling facilitates identifying and quantifying metabolic changes within a biological system in response to various stimuli, such as internal factors like genetic alterations and external factors like pathogen interactions^12^. By gaining insights into the metabolic changes associated with resistance, appropriate strategies can be developed to tackle the *Ganoderma* problem in oil palm plantations in the future.

Proton nuclear magnetic resonance (¹H NMR) spectroscopy is an advanced technique in metabolomic profiling that has been utilized in previous studies to investigate differences in oil palm resistance to *Ganoderma*^13,14^. Unlike other spectrometry techniques, ¹H NMR is nondestructive, unbiased, easily quantifiable, and requires minimal or no chromatographic separation, sample treatment, or chemical derivatization, while also enabling the routine identification of novel compounds^15^. In this study, we applied untargeted metabolomics to analyze root tissues from resistant, susceptible, and control oil palm seedlings challenged with *Ganoderma boninense*. Root tissue was selected due to its direct contact with the pathogen, which is critical for early-stage infection. The aim was to identify metabolite signatures associated with resistance, enabling the discovery of potential biomarkers for early screening. These findings are expected to support breeding programs by facilitating the selection of resistant genotypes and enhancing our understanding of the metabolic mechanisms underlying oil palm defense.

## Results

### ^1^H NMR metabolites profile of oil palm root tissues infected with *Ganoderma*

The^1^H NMR profiling of oil palm root tissues at the nursery stage identified a total of fifty-three (53) metabolites across three sample groups: resistant, susceptible, and control. Specifically, 35 metabolites were detected in resistant root tissues, 39 in susceptible roots, and 44 in control root tissues (Supplementary Table 1).

**Fig. 1:**
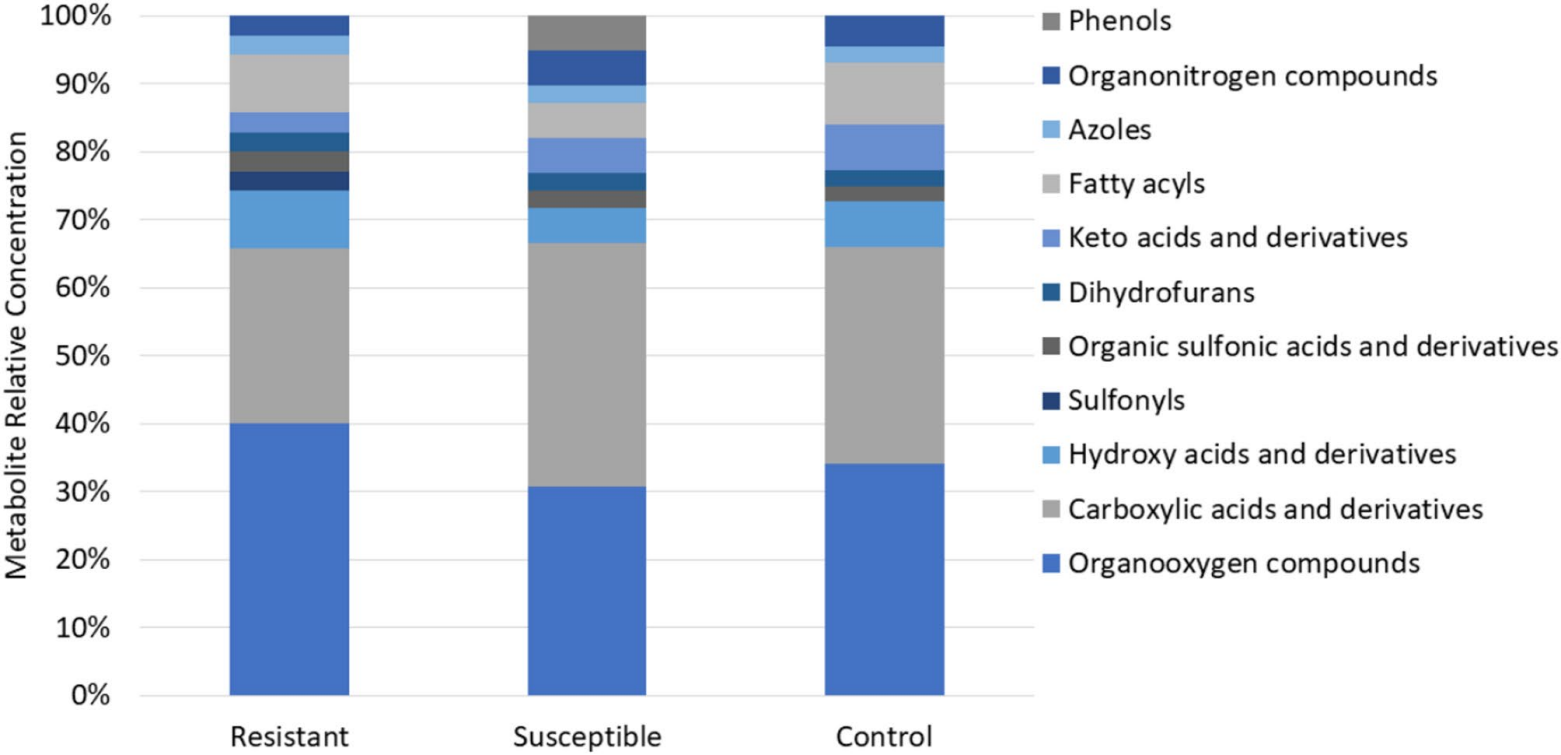
The relative concentration of metabolites in resistant, susceptible, and control root tissues from oil palm seedlings. Each compound class was expressed as a relative percentage across the various resistance categories.

These metabolites were categorized through enrichment analysis into 11 primary classes, including organooxygen compounds (galactitol, glycerol, glyceric acid, myo-inositol, D-sorbitol, D-gluconic acid, D-fructose, glycogen, 2-propanol, threonic acid, acetone, L-arabitol, propylene glycol, threitol, xylitol); carboxylic acids and derivatives (betaine, glycine, guanidoacetic acid, fumaric acid, L-alanine, L-proline, L-isoleucine, L-serine, methylmalonic acid, propionate, sarcosine, 2-aminobutyric acid, aminoadipic acid, N-acetylglycine, L-leucine, malonate, L-valine, trans-aconitic acid); hydroxy acids and derivatives (glycolic acid, lactate, alpha-hydroxyisobutyric acid); sulfonyls (dimethyl sulfone); organic sulfonic acids and derivatives (taurine); dihydrofurans (ascorbic acid); keto acids and derivatives (2-oxobutyrate, 2-oxoisovalerate, acetoacetate); fatty acyls (adipic acid, isovaleric acid, beta-hydroxyisovaleric acid, azelaic acid, sebacic acid, valerate); azoles (allantoin); organonitrogen compounds (ethanolamine, TMAO); and phenols (homovanillic acid, pyrocatechol). The results showed that organooxygen compounds and carboxylic acids and derivatives were the dominant classes across all samples associated with disease resistance (Fig. 1).

This research identified distinct relative metabolites compared to previous studies on^1^H NMR profiling of oil palm resistance to *Ganoderma*. In earlier research,^1^H NMR profiling of oil palm leaf tissues infected with *Ganoderma* revealed 11 main classes of organic compounds, including benzamides, fatty alcohols, organic dicarboxylic acids, alcohols, polyols, sulfonic acids, cholines, furanones, alkanolamines, fatty acids and conjugates, amino acids and peptides, and monosaccharides^14^. Meanwhile,^1^H NMR profiling of stem tissues from oil palms infected with *Ganoderma* identified 10 main classes of organic compounds: polyketides, nucleic acids, organooxygen compounds, fatty acyls, organonitrogen compounds, organoheterocyclic compounds, benzenoids, carbohydrates, and organic acids^13^.

### Classification and identification of metabolites in oil palm seedling roots

**Fig. 2:**
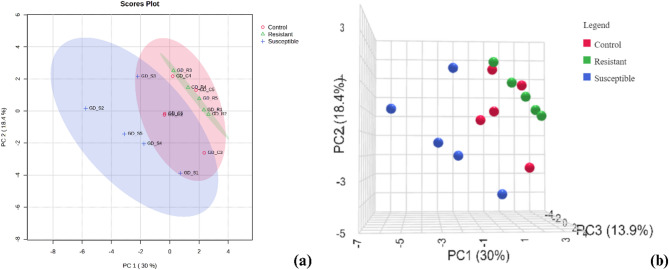
Visualization of (**a**) 2D PCA and (**b**) 3D PCA of metabolites in resistant, susceptible, and control root tissues from oil palm seedlings.

Multivariate data analysis using PCA (2D and 3D) effectively distinguished the metabolite profiles of resistant, susceptible, and control root tissues in oil palm seedlings, as shown in Fig. 2, with PC1, PC2, and PC3 explaining 62.3% of the variation. The analysis clearly separated resistant palms from susceptible ones. Resistant and control roots were relatively grouped together, as neither showed symptoms of *Ganoderma* infection, in contrast to the susceptible palms, which exhibited disease symptoms, resulting in altered metabolite profiles. Consistent with these findings, PCA analysis of NMR data by Rahmadi et al.^14^ also successfully differentiated resistant and susceptible palms. Supporting data (Supplementary Fig. 1) from pairwise 2D PCA comparisons between resistant vs. susceptible, resistant vs. control, and susceptible vs. control showed similar patterns. Resistant and control root samples were closely clustered, whereas resistant and susceptible ones formed distinctly separate groups.

**Fig. 3:**
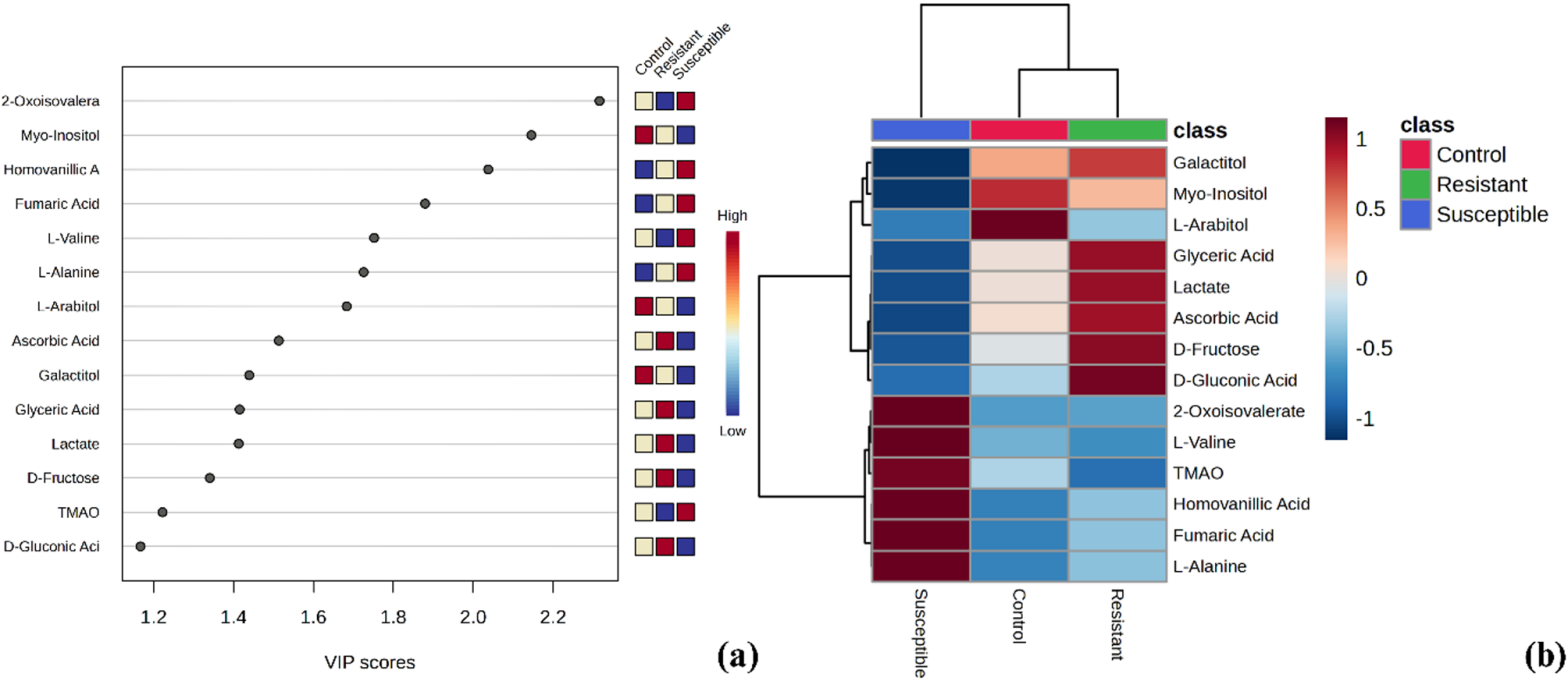
(**a**) PLS-DA analysis result of metabolites in resistant, susceptible, and control root tissues from oil palm seedlings. (**b**) Heatmap visualization of metabolites in resistant, susceptible, and control root tissues from oil palm seedlings. Up-regulated metabolites were represented in red, while down-regulated metabolites were shown in blue.

PLS-DA analysis was conducted to identify significant metabolites differentiating resistant, susceptible, and control root tissues. Fourteen metabolites with VIP scores > 1 were identified as significant (Fig. 3a). The metabolites included 2-oxoisovalerate, myo-inositol, homovanillic acid, fumaric acid, L-valine, L-alanine, L-arabitol, ascorbic acid, galactitol, glyceric acid, lactate, D-fructose, TMAO, and D-gluconic acid. Notably, three metabolites (2-oxoisovalerate, myo-inositol, and TMAO) were concurrently identified in the PLS-DA findings of Rahmadi et al.^14^. Furthermore, four metabolites (D-gluconic acid, L-arabitol, ascorbic acid, and D-fructose) were also found to overlap with the significant metabolite profiles reported by Pancoro et al.^13^ in their investigation of oil palm responses to *Ganoderma* infection.

Fourteen significant metabolites identified from the PLS-DA analysis were further examined using a heatmap to visualize the varying concentrations of each metabolite among resistant, susceptible, and control root tissues in response to *Ganoderma*. The heatmap results mirrored the PCA findings, with resistant roots clustering with control roots (Fig. 3b). Several metabolites, such as glyceric acid, lactate, ascorbic acid, D-fructose, and D-gluconic acid, were up-regulated in resistant roots, while these metabolites were down-regulated in susceptible roots. In accordance with Pancoro et al.^13^, these metabolites were up-regulated in healthy oil palm seedlings compared to those severely affected by *Ganoderma*. In contrast, 2-oxoisovalerate, L-valine, TMAO, homovanillic acid, fumaric acid, and L-alanine were down-regulated in resistant roots compared to susceptible roots.

In this study, OPLS-DA was utilized to compare two resistance categories, complementing the PLS-DA analysis, which identified significant metabolites across all resistance levels. The analysis (Supplementary Fig. 2) identified 14 significant metabolites between resistant and susceptible samples, 18 significant metabolites between resistant and control samples, and 16 significant metabolites between susceptible and control samples, all with a VIP score > 1.0.

### Biomarkers and pathway analysis for oil palm resistance to *Ganoderma*

**Fig. 4:**
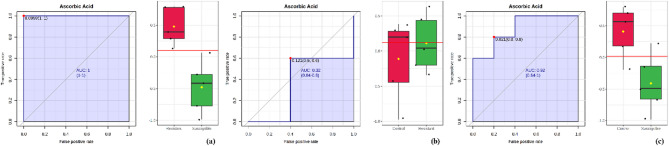
ROC curve and Boxplot of ascorbic acid levels in (**a**) resistant, (**b**) susceptible, and (**c**) control root tissues from oil palm seedlings.

Biomarker identification plays a crucial role in the early detection of diseases^22^. In this study, OPLS-DA and ROC curve analysis were utilized to identify oil palm metabolites capable of distinguishing between resistance and susceptibility to *Ganoderma*. The OPLS-DA results, along with a ROC curve (AUC) = 1, indicate that these metabolites have the potential to serve as reliable biomarkers. An AUC score 1 signifies that the classifier can accurately differentiate between positive and negative classes, with no false positives^13,14^. Several metabolites were identified as significant biomarkers (Table 1). Ascorbic acid, D-gluconic acid, D-fructose, and 2-oxoisovalerate exhibited significant differences in resistant roots compared to susceptible roots, suggesting their potential as biomarkers for screening *Ganoderma*-resistant oil palms. These metabolites are crucial in distinguishing resistance levels, providing valuable insights for breeding programs. To further illustrate these differences, box plots were generated, highlighting the significant variation in ascorbic acid concentrations among resistant, susceptible, and control root tissues (Fig. 4).

**Table 1:** List of metabolite compounds identified through OPLS-DA analysis of oil palm root tissues against *G. boninense*. Metabolites marked with an asterisk (*) have an AUC value of 1, indicating their potential as candidate biomarkers.

No.	Metabolite	VIP score of potential biomarker		
		Resistant compared to susceptible	Resistant compared to control	Susceptible compared to control
1	Ascorbic acid	1.55*	0.62	1.67
2	d-Gluconic acid	1.66*	1.74	1.38
3	d-Fructose	1.58*	0.93	1.48
4	2-Oxoisovalerate	1.78*	0.47	2.25
5	Myo-Inositol	1.22	1.45	1.74*

**Fig. 5:**
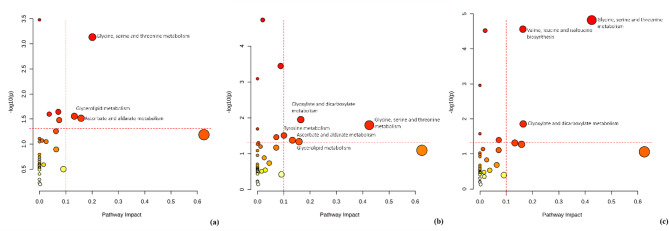
Pathway analysis of metabolites in (**a**) resistant, (**b**) susceptible, and (**c**) control root tissues from oil palm seedlings (p-value < 0.05 and pathway impact > 1). The horizontal dotted line represents the threshold for statistical significance (p-value), while the vertical line denotes the cutoff for pathway impact scores.

Pathway analysis was performed to determine the metabolic pathways associated with oil palm resistance to *Ganoderma*. Utilizing the KEGG database for pathway enrichment analysis (p-value < 0.05 and pathway impact > 0.1), the results revealed that basal stem rot (BSR) disease affects distinct metabolic pathways in resistant, susceptible, and control root tissues. Notably, the glycine, serine, and threonine metabolism pathways were consistently present across all three root conditions (Fig. 5). However, ascorbate and aldarate metabolism, as well as glycerolipid metabolism, were detected only in resistant and susceptible roots.

## Discussion

The analysis of 1 H NMR spectra of oil palm root tissues revealed key differences in the metabolic profiles among resistant, susceptible, and control oil palm seedlings. Enrichment analysis highlighted that sulfonyl compounds were exclusively detected in resistant roots. These compounds are known as plant immune-priming agents that enhance disease resistance^23^. Sulfonyl compounds trigger the biosynthesis of salicylic acid (SA), which strengthens the plant’s defense against pathogens, including fungi, bacteria, and viruses^24^. Among the sulfonyl compounds identified, dimethyl sulfone was notably up-regulated in resistant roots (Supplementary Fig. 3). Supporting this finding, dimethyl sulfone has demonstrated antifungal activity against *Botrytis cinerea* in a separate study^25^.

In contrast, phenolic compounds were exclusively detected in susceptible roots infected with *Ganoderma*. Plants under stress or infection often increase the production of phenolic compounds as a response to cellular damage, aiding in defense against pathogens^26^. The elevated phenol levels in susceptible plants are believed to indicate that the plants are attempting to respond to infection or stress, but their defense mechanisms are not yet strong enough to effectively combat the pathogens. The phenolic compounds up-regulated in susceptible plants were identified as homovanillic acid and pyrocatechol (Supplementary Fig. 3). The role of homovanillic acid in plants is not well understood, though it acts as a potential antioxidant in animal cells^27^. Pyrocatechol, on the other hand, plays a role in protecting plants against pathogens and contributes to nitrogen detoxification^28^. In tomatoes, pyrocatechol has been shown to prevent disease symptoms after infection by *Fusarium oxysporum*^29^.

The grouping of resistant and control root tissues in the PCA analysis suggests that resistant palms maintain a metabolite profile closer to that of healthy, uninfected plants. This implies that resistant palms possess innate or early-induced defenses that minimize metabolic disruption during Ganoderma infection, thereby preventing the drastic biochemical changes observed in susceptible plants. According to Zhu et al.^30^, plant immunity inducers can prime plant defense mechanisms and significantly reduce the occurrence and severity of plant diseases. Moreover, resistant plants often maintain homeostasis by producing specific defense-related metabolites that inhibit pathogen growth. Similarly, Jian et al.^31^ reported that plants produce defense molecules that directly suppress pathogen growth and development through both constitutive and inducible mechanisms.

PLS-DA analysis revealed key metabolites that are significantly different across the resistance categories. Moreover, the heatmap results further support these findings, showing a pattern where metabolites up-regulated in resistant roots are consistent with known defense-related compounds. This up-regulation suggests that these metabolites play a crucial role in strengthening the plants defense against *Ganoderma* infection, while their down-regulation in susceptible roots implies a weakened or compromised defense mechanism. Furthermore, according to Anjali et al.^32^, up-regulated metabolites in plants due to pathogen attacks contribute to the formation of a robust defense system, which boost plant survival. In addition, several studies have shown that these defense-related metabolites not only act as direct antimicrobial agents but also serve as signaling molecules that activate further immune responses^33–37^.

Biomarker analysis identified ascorbic acid, D-gluconic acid, D-fructose, and 2-oxoisovalerate as significant biomarkers with potential for screening resistant palms, as they play essential roles in plant defense mechanisms. Ascorbic acid, particularly, is well known for protecting plant cells from oxidative stress induced by pathogen attacks^38^. Furthermore, in vitro studies have demonstrated that the growth of the soil-borne hemibiotrophic fungus *Macrophomina* is significantly inhibited in media containing ascorbic acid compared to those without it, suggesting its direct antifungal properties^35^. Similarly, D-gluconic acid exhibits antifungal activity and has potential as a biocontrol agent, as it disrupts fungal growth and enhances plant resistance^39,40^. In addition, cultivating *Rhodosporidium paludigenum* with gluconic acid improved its ability to control green mold infections in citrus fruit^34^. Moreover, D-fructose is recognized as a key regulatory molecule in plant defense responses to fungal pathogens^33^. Notably, elevated sugar levels in plant tissues enhance the plant’s immune response against fungal pathogens^41^. Unfortunately, the specific role of 2-oxoisovalerate in plant defense against pathogens remains poorly understood. Therefore, further research is needed to elucidate its potential function in plant immunity and stress responses.

Pathway analysis revealed the involvement of glycine, serine, and threonine metabolism pathways in all three root conditions (resistant, susceptible, and control root tissues). This finding aligns with previous studies by Pancoro et al.^13^ and Rahmadi et al.^14^. These pathways are fundamental to protein synthesis and play a crucial role in plant responses to environmental stress^42^. Their significance has also been demonstrated in other studies, such as mandarin fruit responses to cyclic lipopeptides from *Bacillus subtilis*^43^. Furthermore, several proteins within the glycine-rich protein (GRP) superfamily have been identified as key players in cellular signaling and stress responses in plants^44^.

Interestingly, both resistant and susceptible roots infected with *Ganoderma* showed activation of the ascorbate and aldarate metabolism and glycerolipid metabolism pathways. Meanwhile, in the control treatment, these metabolic pathways were not detected. This indicates that the ascorbate and aldarate metabolism and glycerolipid metabolism pathways are only active when *Ganoderma* infection occurs. Based on Fick et al.^45^, some genes involved in plant immune response activation are triggered during pathogen infection. The activation of these metabolic pathways suggests a potential role in the plant’s defense mechanisms, possibly linked to oxidative stress regulation and lipid signaling.

Ascorbate and aldarate metabolism is crucial for protecting cells from oxidative damage, with ascorbic acid acting as a potent antioxidant that neutralizes reactive oxygen species (ROS) generated during pathogen invasion. Studies have shown that plants with lower ascorbate levels are more susceptible to fungal infections^46,47^. Glycerolipid metabolism, which regulates lipid signaling and ROS metabolism, plays a vital role in plant defense. Lipids and lipid-derived compounds can exhibit antimicrobial activity and contribute to plant defense against pathogens^48–50^. Furthermore, detecting fatty acids in root tissues suggests a potential activation of glycerolipid metabolism, which is critically involved in membrane remodeling during pathogen response^51^.

These findings imply that *Ganoderma* infection triggers specific metabolic adjustments that could be crucial for the plant’s adaptive response to pathogen stress. The activation of these pathways highlights the dynamic nature of the plant’s defense mechanisms. Overall, this study highlights several key metabolites and pathways differentially regulated in resistant versus susceptible oil palm seedlings, offering insights into the underlying mechanisms of Ganoderma resistance and potential biomarkers for breeding and early screening.

## Conclusion

¹H NMR successfully identified metabolites that distinguish between oil palm seedlings resistant and susceptible to basal stem rot (BSR) caused by *Ganoderma*. Multivariate data analysis effectively differentiated resistant and susceptible palms, revealing that ascorbic acid, D-gluconic acid, D-fructose, and 2-oxoisovalerate could serve as potential biomarkers for screening resistant palms. Pathway analysis indicated that both resistant and susceptible roots infected with *Ganoderma* exhibited similar metabolic pathways, particularly ascorbate and aldarate metabolism, as well as glycerolipid metabolism, which play crucial roles in oil palm defense mechanisms against *Ganoderma* infection. Furthermore, these findings enhance our understanding of disease resistance mechanisms and can facilitate the breeding of new oil palm varieties with improved resistance to *Ganoderma*.

## Methods

### Sample collection

**Fig. 6:**
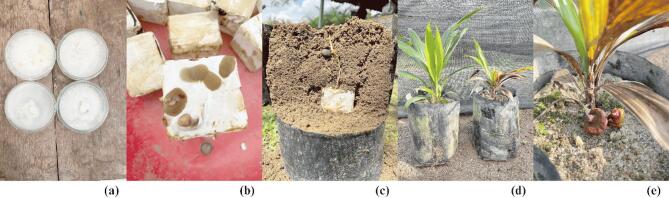
(**a**) *Ganoderma boninense* isolate. (**b**) RWB infected with *Ganoderma*. (**c**) Sitting germinated seed technique for *Ganoderma* inoculation in oil palm seedlings. (**d**) Comparison of resistant and susceptible oil palms after six months of infection (**e**) Fruiting body of *Ganoderma* on susceptible oil palm.

The oil palm root tissues used in this study were collected from an oil palm nursery at the Indonesian Oil Palm Research Institute (IOPRI) in Marihat, Simalungun Regency, North Sumatra, Indonesia. Three categories of samples were used in this study: resistant, susceptible, and control roots. Six-month-old oil palm seedlings, both resistant and susceptible that had been previously inoculated with *Ganoderma* were used. Seedling infection was conducted using the rubber wood block (RWB) method, with inoculum prepared by growing isolates on blocks of sterilized rubber wood. These blocks were then placed beneath germinating seedlings growing in soil-filled polybags (sitting germinated seed technique) (Fig. 6). The resistant seedlings, or healthy plants, were identified based on the absence of mycelium on the roots or necrosis on the basal stems and leaves. Susceptible seedlings were identified by the presence of mycelium on the roots, necrosis on more than 25% of the basal stem, and the appearance of *Ganoderma* fruiting bodies. Additionally, six-month-old control seedlings that had not been treated with *Ganoderma* infection were included. Each treatment had five individuals as biological replicates, totaling 15 oil palm seedlings tested using ¹H NMR.

### Metabolite extraction

The roots of oil palm seedlings were thoroughly washed and then oven-dried at 60 °C for 5 days or until a constant weight was achieved. One gram of the dried root sample was ground using a mortar and liquid nitrogen, then dissolved in 30 mL of methanol^16^. The mixture was subjected to maceration on a shaker at 115 rpm overnight. Ultrasonic extraction was subsequently carried out at 65 °C for 30 min, followed by centrifugation at 3500 rpm for 20 min. The supernatant was collected and evaporated with a rotary evaporator to yield the crude extract.

### ^1^H NMR profiling

This research employed an untargeted metabolomics approach based on ¹H NMR^17^. A total of 50 ± 0.1 mg of the crude extract was accurately weighed, and 1.0 mL of methanol-d4 (Merck-Sigma, USA) containing 0.001% trimethylsilyl propionic acid-d4 sodium salt (TMSP) was added as an internal standard. The mixture was vortexed for 60 s, sonicated for 20 min, and then centrifuged at 10,000 rpm for 5 min. The supernatant was placed into an NMR tube for ¹H NMR profiling. The analysis was conducted using a JEOL JNM-ECZR NMR spectrometer (Tokyo, Japan) operating at a proton frequency of 500 MHz, with water signal suppression applied at a chemical shift of 4.89 ppm.

### Preprocessing data

The NMR spectra were processed using Mestrenova software version 14.3, which included Fourier transformation, phase correction, and baseline adjustment to ensure data accuracy^18^. The spectra were calibrated using the TMSP-2,2,3,3-d4 signal at δ 0.0 ppm as the reference standard. Metabolite identification and quantification from the ¹H NMR spectra were performed using the ASICS package in the R programming language, which enables automated spectral deconvolution for precise metabolite profiling^19^.

### Multivariate statistical analysis

The normalized NMR data matrix was analyzed using MetaboAnalyst 5.0 ^20^, with Pareto scaling applied prior to statistical and multivariate analyses. The analyses included principal component analysis (PCA), partial least squares discriminant analysis (PLS-DA), and orthogonal projections to latent structures discriminant analysis (OPLS-DA) to classify and differentiate samples based on multidimensional data. Additionally, heatmap visualization was used to highlight metabolite variations across different sample groups. Variable importance in projection (VIP) scores and receiver operating characteristic (ROC) curves were employed to identify potential biomarkers associated with *Ganoderma* resistance. Pathway analysis, incorporating pathway enrichment techniques, was performed to identify critical pathways linked to resistance mechanisms. Significant metabolic pathways were determined based on the p-value (< 0.05) and impact value score (> 0.1)^13,21^. The Kyoto Encyclopedia of Genes and Genomes (KEGG) database was used as a metabolic pathway identification reference.

## Electronic supplementary material

Below is the link to the electronic supplementary material.

Supplementary Material 1

Supplementary Material 2

Supplementary Material 3

Supplementary Material 4

## Data Availability

The datasets generated and analyzed during this study are available from the corresponding author upon reasonable request.
