# Supplementary material for: 1H NMR metabolomic profiling of resistant and susceptible oil palm root tissues in response to Ganoderma boninense at the nursery stage
Source: Sci Rep. 2025 May 14;15:16784. doi: 10.1038/s41598-025-01691-y (PMC12078656; doi:10.1038/s41598-025-01691-y)
Supplement: Supplementary file 1 — Supplementary Material 1 [file 41598_2025_1691_MOESM1_ESM.pdf]

Supplementary Figure 3

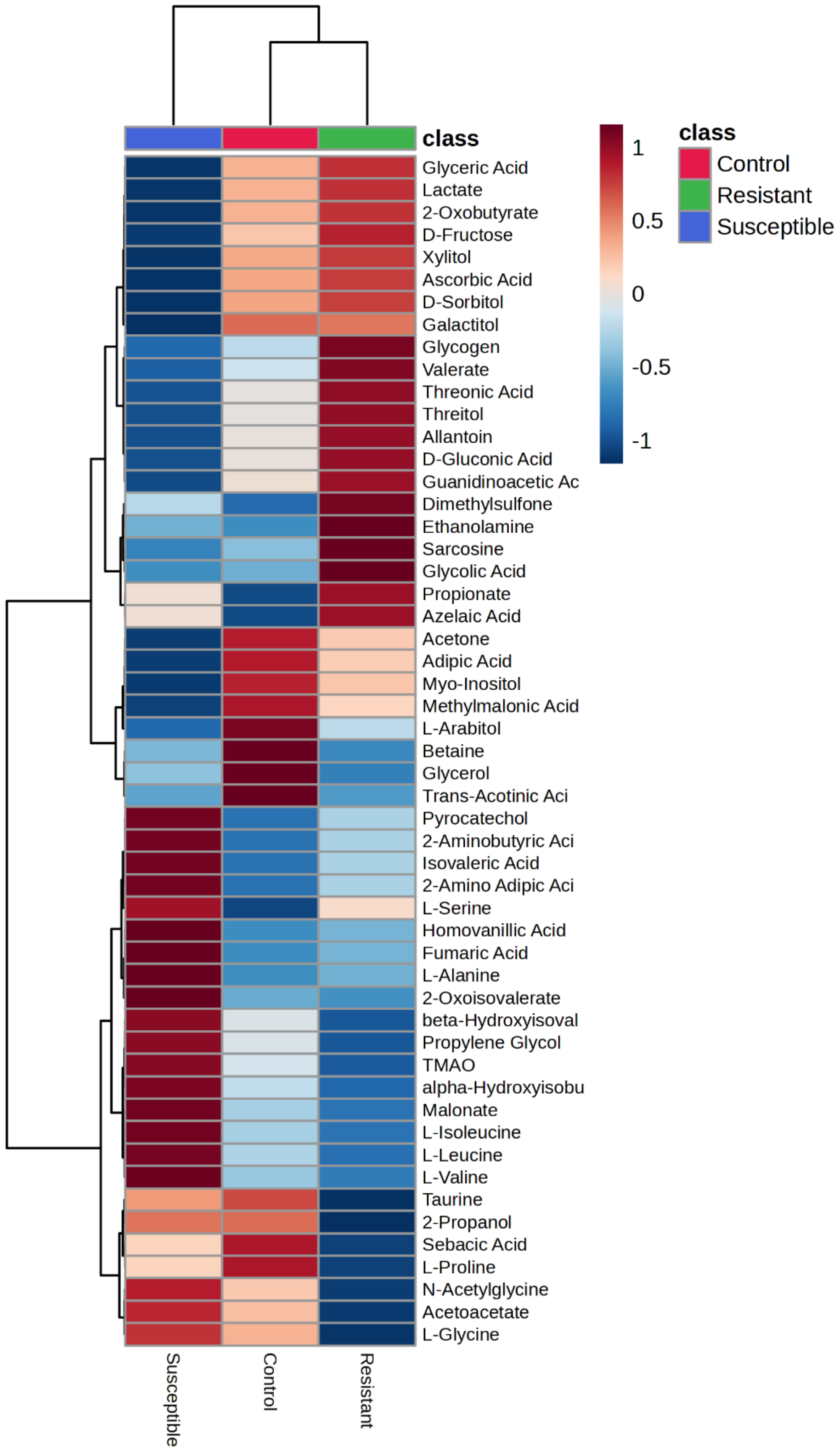

Supplementary Figure 3. Heatmap visualization of top 25 metabolites in resistant, susceptible, and control root tissues from oil palm seedlings. Upregulated metabolites were represented in red, while downregulated metabolites were shown in blue.
