# Supplementary material for: 1H NMR metabolomic profiling of resistant and susceptible oil palm root tissues in response to Ganoderma boninense at the nursery stage
Source: Sci Rep. 2025 May 14;15:16784. doi: 10.1038/s41598-025-01691-y (PMC12078656; doi:10.1038/s41598-025-01691-y)
Supplement: Supplementary file 2 — Supplementary Material 2 [file 41598_2025_1691_MOESM2_ESM.pdf]

Supplementary Figure 2

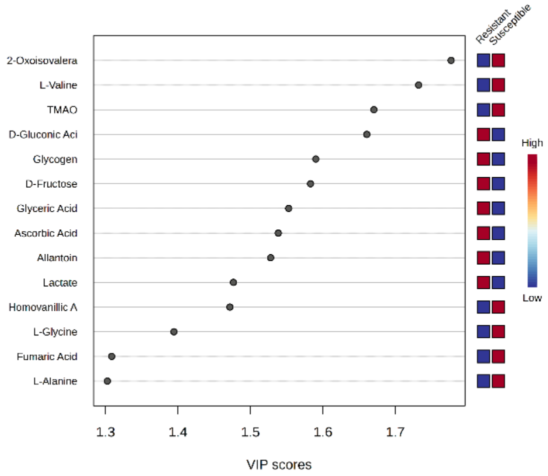

(a)

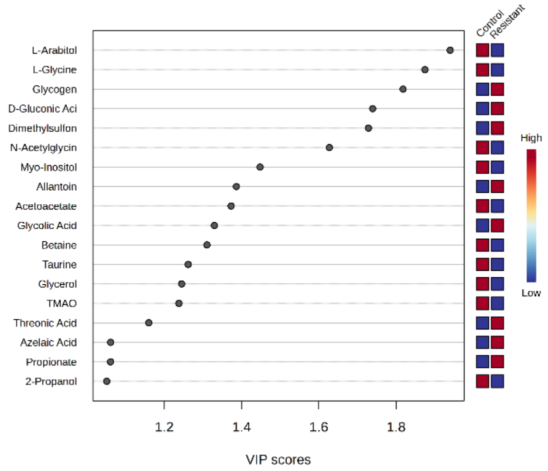

(b)

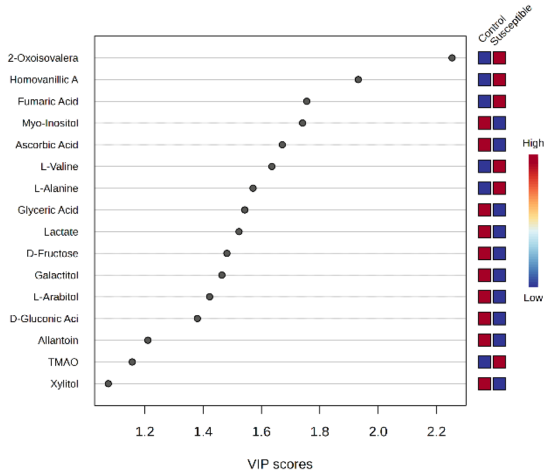

(c)

Supplementary Figure 2. Results of the analysis of significant compounds using OPLS-DA on (a) the comparison between resistant and susceptible samples, (b) the comparison between resistant and control samples, and (c) the comparison between susceptible and control samples, illustrating the differences in oil palm resistance to *Ganoderma*.
