# Supplementary material for: 1H NMR metabolomic profiling of resistant and susceptible oil palm root tissues in response to Ganoderma boninense at the nursery stage
Source: Sci Rep. 2025 May 14;15:16784. doi: 10.1038/s41598-025-01691-y (PMC12078656; doi:10.1038/s41598-025-01691-y)
Supplement: Supplementary file 3 — Supplementary Material 3 [file 41598_2025_1691_MOESM3_ESM.pdf]

Supplementary Figure 1

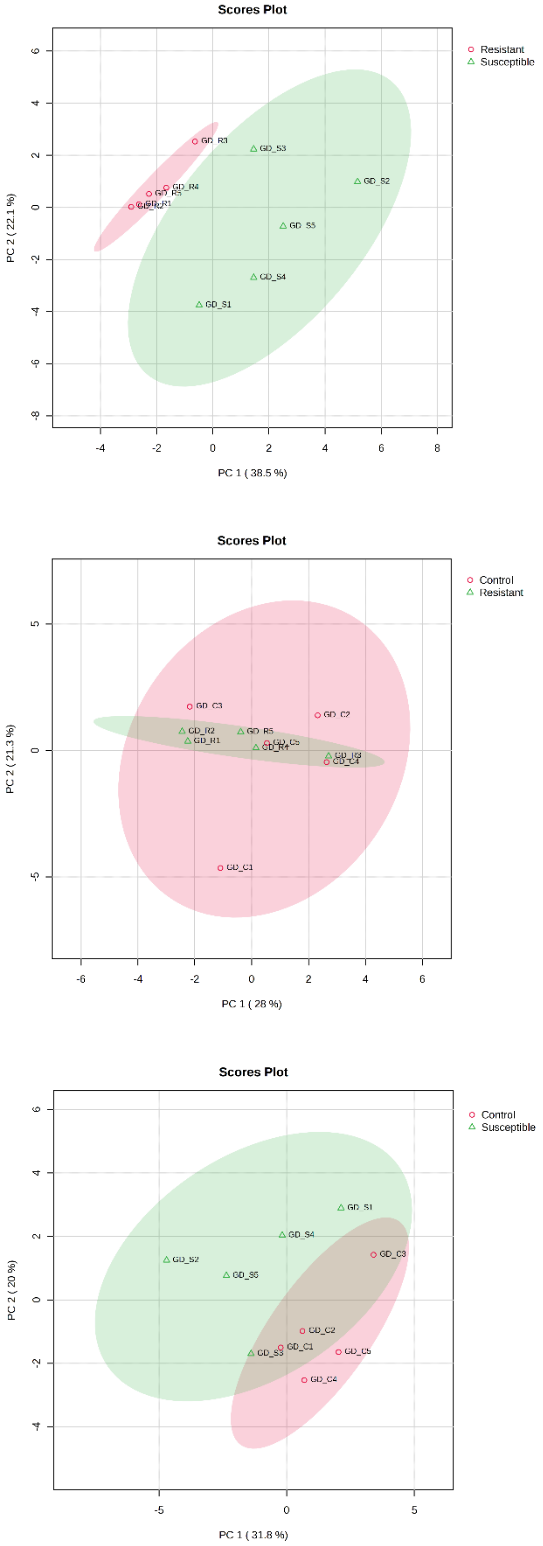

(a)

(b)

(c)

Supplementary Figure 1. The 2D PCA visualization results: (a) comparison between resistant and susceptible samples, (b) comparison between resistant and control samples, and (c) comparison between susceptible and control samples, illustrating the differences in oil palm resistance to *Ganoderma*.
